# Expression of concern: Insulin loaded iron magnetic nanoparticle–graphene oxide composites: synthesis, characterization and application for *in vivo* delivery of insulin

**DOI:** 10.1039/d4ra90136c

**Published:** 2024-12-06

**Authors:** Kostiantyn Turcheniuk, Manakamana Khanal, Anastasiia Motorina, Palaniappan Subramanian, Alexandre Barras, Vladimir Zaitsev, Victor Kuncser, Aurel Leca, Alain Martoriati, Katia Cailliau, Jean-Francois Bodart, Rabah Boukherroub, Sabine Szunerits

**Affiliations:** a Institut de Recherche Interdisciplinaire (IRI, USR CNRS 3078), Université Lille 1 Parc de la Haute Borne, 50 Avenue de Halley, BP 70478, 59658 Villeneuve d'Ascq France Sabine.Szunerits@iri.univ-lille1.fr; b Department of Fine Organic Synthesis, Institute of Bioorganic Chemistry and Petrochemistry NAS of Ukraine 1 Murmanska Str. 02660 Kiev Ukraine; c Taras Shevchenko University 60 Vladimirskaya str. Kiev Ukraine; d National Institute of Materials Physics Atomistilor 105 bis, 077125 Magurele Romania; e EA 4479, IFR 147, Université Lille 1 59658 Villeneuve d'Ascq France

## Abstract

Expression of concern for ‘Insulin loaded iron magnetic nanoparticle–graphene oxide composites: synthesis, characterization and application for *in vivo* delivery of insulin’ by Kostiantyn Turcheniuk *et al.*, *RSC Adv.*, 2014, **4**, 865–875, https://doi.org/10.1039/C3RA46307A.

The Royal Society of Chemistry is publishing this expression of concern in order to alert readers that concerns have been raised regarding the reliability of the data. The Royal Society of Chemistry has asked the University of Lille to investigate this matter. An expression of concern will continue to be associated with the article until we receive conclusive evidence regarding the reliability of the reported data.

Laura Fisher

5th November 2024

Executive Editor, *RSC Advances*

